# Estimating health related quality of life effects in vitiligo. Mapping EQ-5D-5 L utilities from vitiligo specific scales: VNS, VitiQoL and re-pigmentation measures using data from the HI-Light trial

**DOI:** 10.1186/s12955-023-02172-4

**Published:** 2023-08-10

**Authors:** Rabiah Begum, Ralph Crott, Reynaldo Martina, Eleni M. Loizidou, Iftekhar Khan

**Affiliations:** 1 Department of Population Health Sciences, University of Leicester, Leicester, UK; 2 IRSS, Catholic University of Louvain, Brussels, 1200 Belgium; 3 Open University UK, Milton Keynes, UK; 4 Biobank.cy, Center of Excellence in Biobanking and Biomedical Research, University of Cyprus, Nicosia, Cyprus; 5 Warwick Clinical Trials Unit, Warwick Medical School, University of Warwick, Coventry, UK

**Keywords:** Mapping, Dermatology, Vitiligo, EQ-5D, EuroQoL, VNS, VitiQoL, Re-pigmentation

## Abstract

**Background:**

Vitiligo is reported to affect 2% of the world’s population and has a significant impact on health related quality of life (HRQoL). The relationship between HRQoL and clinical outcomes used in vitiligo require further examination. Mapping condition specific measures of HRQoL: vitiligo specific quality of life instrument (VitiQoL), vitiligo noticeability scale (VNS) and vitiligo re-pigmentation scores (RPS) to the EQ-5D have not yet been reported.

**Methods:**

Data collected from a randomised clinical trial (HI-Light) in vitiligo was used to develop mapping algorithms for the EQ-5D-5 L and the relationship between HRQoL, clinical outcomes and EQ-5D were investigated. Two EQ-5D-5 L value sets (Van Hout and Alava) using linear and non-linear models were considered. Logistic regression models were used to model the probability of vitiligo noticeability (VNS) in terms of RPS, EQ-5D and VitiQoL scores.

**Results:**

Mapping from RPS appeared to perform better followed by VNS for the Alava crosswalks using polynomial models: Mean observed vs. predicted utilities of 0.9008 (0.005) vs. 0.8984 (0.0004) were observed for RPS. For VNS, mean observed vs. predicted utilities of 0.9008 (0.005) vs. 0.8939 (0.0003) were observed. For VitiQoL, mean observed vs. predicted utilities of 0.9008 (0.005) vs. 0.8912 (0.0002) were observed. For patients with the least re-pigmentation (RPS < 25%), a Total VitiQoL score of about 20 points gives around an 18% chance of vitiligo being no longer or a lot less noticeable.

**Conclusion:**

The algorithm based on RPS followed by VNS performed best. The relationship between effects from vitiligo specific HRQoL instruments and clinical RPS was established allowing for plausible clinically relevant differences to be identified, although further work is required in this area.

**Supplementary Information:**

The online version contains supplementary material available at 10.1186/s12955-023-02172-4.

## Introduction

Vitiligo affects 2% of the world’s population with significant impact on health related quality of life (HRQoL) [1]. Responses from the EQ-5D (EQ-5D-5 L or EQ-5D-3 L) are converted to ‘health utilities’, required by various health technology assessment (HTA) bodies, to assess the value of health technologies [2, 3]. In particular, the EQ-5D is used to estimate gain (or loss) in quality-adjusted life years (QALYs) when determining cost-effectiveness (cost per QALY gained). The EQ-5D, however, is not always collected. This can happen where condition specific HRQoL measures (CSM) are considered more important, or, when EQ-5D is considered irrelevant to (payer) decision making [3, 4]. Moreover, effects from some HRQoL measures have not been evaluated in detail when anchored against clinical outcomes such as re-pigmentation scores (RPS).

When EQ-5D utilities are not collected, alternative methods such as ‘mapping’ may be used [4–6]. Mapping involves the development of an algorithm through statistical methods used to predict utilities from CSMs collected in a *different* trial [7, 8]. In practice, an algorithm is published so that patient level utilities can be computed and used to generate QALYs. Mapped utilities can therefore be superior to those determined from different populations. The benefits and limitations of mapping have been reported elsewhere [8–11].

There are several CSMs used for vitiligo patients. The dermatology life quality index (DLQI) is a 10-item validated questionnaire to measure how much the skin problem has affected patients [12]. The European Academy of Dermatology and Venerology Task Force (EADV) evaluated the use of HRQoL instruments in vitiligo, noting the DLQI as the most ‘frequently’ used instrument [13]. However, the EADV also noted some items in the DLQI are irrelevant for most patients with vitiligo (e.g., itching). Consequently, other vitiligo specific HRQoL instruments may be more relevant: the Vitiligo Impact Scale (VIS) [14], the Vitiligo Area Scoring Index (VASI) [15], Vitiligo specific quality of life instrument (VitiQoL) [16] and Vitiligo Noticeability Scale (VNS) [17].

The VIS [14] is a validated instrument with a key limitation being lack of generalizability because responses to the questions revolve around ethno-centric aspects of vitiligo in India [14]. The VitiQoL is a 16-item instrument where participants (over the past month) rate their vitiligo using a 7-point scale (“Not at all” to “All of the time”) [16]. The VNS measures treatment success, over a 5-point scale [17]. In practice, an image (photograph) is shown to a patient prior to treatment (baseline) and at several points, post treatment. VNS scores of between 3 and 5 suggest good outcomes.

Currently, no mapping algorithm exists in a vitiligo specific population, although several are published in psoriasis or atopic dermatitis [18–24]. These algorithms used conversions (crosswalks) between EQ-5D-3 L and EQ-5D-5 L using methods (Van Hout) [25] no longer fully supported by the NICE DSU [26]. None of these algorithms used data from randomized controlled trials (RCT); and none relate utilities to clinical outcomes (such as percent re-pigmentation). Consequently, a vitiligo specific mapping algorithm that reflects current methods is needed. There is also a need to identify the relationship between effects from vitiligo specific HRQoL, generic HRQoL and clinical outcomes.

We use data from the HI-Light trial [1], a RCT in adults and children, aged ≥ 5 years; with vitiligo affecting < 10% of skin, to develop new mapping algorithms from VNS, VitiQoL and re-pigmentation scores (RPS). The HI-Light trial took place in the United Kingdom (UK). The HI-Light protocol was approved by the East Midlands-Derby Research Ethics Committee (14/EM/1173), MHRA (EudraCT 2014-003473-42) with ISRCTN: 17,160,087.

Utilities from two currently used crosswalks: Van Hout et al., (2012) [25] and Alava et al., (2022) [27] are compared. The findings from this research aim to fill an important knowledge gap allowing health utilities to be derived from vitiligo specific HRQoL instruments as well as examining the relationship between clinical and HRQoL effects.

## Methods

### Data collection

Data were collected from 517 participants (398 adults; 119 children) from the HI-Light trial [1] ; 173 randomised to Topical Corticoid Steroids (TCS); 169 to handheld Narrowband Ultraviolet B (NB-UVB (a form of phototherapy)) and 175 to a combination of potent topical corticosteroid (TCS) + NB-UVB (1:1:1 allocation). Participants (aged > 5 years), had nonsegmental vitiligo (≤ 10% body surface area), and at least one vitiligo patch that had been active in the last 12 months. Data collected at baseline (screening), 9 and 21 months post randomization were used in this analysis. The primary outcome was participant-reported treatment success (‘a lot less noticeable’ or ‘no longer noticeable’) at the target patch of vitiligo, after 9 months of treatment, using the VNS. The full results of the trial have been reported elsewhere [1], where only the combination treatment (TCS + NB-UVB) was statistically superior to TCS (*p* = 0.032).

### HRQoL instruments

#### EQ-5D-5 L

The EQ-5D-5 L is a generic HRQoL measure used in economic evaluations. The 5 L is measured on a 5-point scale for each of the following domains: Mobility, Self-Care, Usual Activities, Pain/Discomfort, and Anxiety/Depression. Scoring the EQ-5D-5 L is well established [2]: each of the scores generate a health state between '11111' (best possible health state) to '55555' (worst possible health state) converted to a score between − 0.594 (worst possible health state) to 1.00 (best possible health state) using the UK (or a country specific) tariff [25]. However, since the scoring (tariff) for the 5 L version for the UK remains to be finalised [28], the Van Hout (VH) crosswalk between the EQ-5D-3 L (3 L) and 5 L is used, which ‘converts’ 5L utilities to 3L utilities as an intermediate step [25]. Reporting of EQ-5D in the HI-Light trial was based on the VH crosswalk [1]. The Alava crosswalk is also used in this analysis for the purpose of ‘converting’ 5L utilities to 3L utilities [27].

#### VNS

The VNS is a patient-reported measure (5-point scale) of vitiligo treatment success [17]. Patients provide responses as: [1] More noticeable, [2] As noticeable, [3] Slightly less noticeable, [4] A lot less noticeable, and [5] No longer noticeable, in relation to vitiligo. Scores of 4 or 5 represent treatment success. VNS scores of 4/5 have been used as primary/secondary outcomes in trials [17]. The relationship between a score of 4 or 5 and clinical outcomes such as RPS has not been previously fully explored.

#### VitiQoL

The VitiQoL is a 16-item HRQoL assessment where patients rate (7-point scale) various aspects of their vitiligo from: “Not at all” to “All of the time” [16]. The total score (range 0 to 90) is derived, with high scores indicating worse HRQoL. No clear clinically relevant effect size is reported.

#### Re-pigmentation measures

Percentage re-pigmentation was assessed using blinded clinician assessment of digital images. Re-pigmentation scores measured in the HI-Light trial were computed for each lesion on the face, hands/feet and ‘other’ body parts. In practice, the total area per lesion is computed at baseline and post baseline as a percentage of the body surface area (BSA) and the differences in these measures between baseline and post-baseline are expressed as a percentage change. Each percentage change falls into one of 4 classification categories: 0–24%, 25–49%, 50–74% and 75–100%. This is equivalent to the vitiligo area score index (VASI) [15]. Re-pigmentation in the trial was assessed on the hands/feet, head/neck (i.e., face) and rest of the body. Mapping was based primarily on combining the data across hands/feet and face as this is where the impact on HRQoL was considered to be greatest.

### Statistical methods

#### Mapping model specification

Mapping was undertaken (by instrument and crosswalk) following commonly accepted methods [7–9, 18–24]. Firstly, observed EQ-5D-5 L responses were converted to utilities (Alava and VH). Secondly, after plotting utilities, several models were considered: Linear, and Non-Linear in a frequentist and Bayesian framework. Thirdly, model performance was based on metrics such as: observed vs. predicted mean utilities and QALY estimates, mean absolute error (MAE), root mean square error (RMSE), Akaike / Bayesian Information criterion (AIC/BIC), Deviance Information Criterion (DIC for Bayesian). The number of health states observed were reported and plots of predicted vs. observed utilities were generated for each model. Covariates such as age were considered in early model selection criteria, however age and gender were found to have no statistical significance when included in the final VitiQoL and VNS models. In addition, a covariate for the location of the lesion (face vs. hands/feet) was included (but subsequently dropped as not statistically significant).

Cross validation methods were used on 50% of the data where data were randomly split into half and models were built with one half of the sample to predict the estimates that could be compared with the observed values in the other half of the sample.

#### Model structure: VitiQoL

Linear models (M1 and M2) for Total VitiQoL score (TVS) and each of the 16 VitiQoL component scores in a frequentist and Bayesian (TVS only) framework (M3) were modelled. For M3: Bayesian Linear Models (BLM), we assume a model of the form:

$$\mathrm{EQ}-5\mathrm D-5{\mathrm L}_{\mathrm{VH}/\mathrm{AL}}=\mathrm\mu\;+\;\upbeta\mathrm{i}\;+\;\gamma\mathrm{j}\;+\;\epsilon\mathrm{j}$$

where β_i_ is the fixed effect for TVS, γ_j_ is the random subject effect ~ N(0,σ_γ_^2^); ϵ_j_ ~ N(0,σ^2^), the random error and µ is the overall mean; we assume further, a non-informative prior for β_i_ such that:

$$\mathrm\pi\left(\upbeta\mathrm{i}\right)\sim\mathrm N\left(0,1\mathrm e^5\right)$$

$${\mathrm\pi\left(\gamma\mathrm{j}\right)\sim\mathrm N\left(0,\;{\mathrm\sigma^2}_{\mathrm\gamma}\right)}$$

The priors on the variance terms σ^2^ and σ^2^_γ_ are assumed to be inverse gamma (IG):

$$\mathrm\pi\left(\mathrm\sigma^2\right)\sim\mathrm{IG}\left(\mathrm\varpi=\;\mathrm{shape}=2.001,\;\mathrm\theta=\mathrm{scale}=1.001\right)$$

$${\pi}\left({\mathrm\sigma^2}_{\mathrm \gamma}\right)\sim\mathrm{IG}\left(\mathrm\varpi=2.001,\;\mathrm\theta=1.001\right)$$

The normal prior on the parameters for the fixed effect (β_i_ for TVS) is assumed to have a large variance suggesting little or no knowledge about the regression parameters β_i_. The priors for the variance terms use the IG with ω and θ for the shape and scale parameters respectively, reflecting lack of knowledge about the variance coefficients. The DIC was used to evaluate model fit as an approximate to the AIC [29]. Hence, three models : M1, M2 and M3 were fitted for VitiQoL.

#### Model structure: VNS and RPS

For VNS and RPS, due to the discrete nature of the categories and initial plots (Supplementary Figure 2), Linear (M4), Non-Linear (M5) and Polynomial regressions (M6) were fitted for RPS and VNS. The non-linear form (M5) of the model is part of the 4 parameter models described in Ratowsky (1990) [30] allowing greater flexibility for curve fitting of conditional mean models:

$$\mathrm{EQ}-5\mathrm D-5{\mathrm L}_{\mathrm{VH}/\mathrm{AL}=}\dots\mathrm\alpha+\mathrm\beta\ast\mathrm{VNS}+\mathrm\gamma\ast\log\left(\mathrm{VNS}+\delta\right)..\left(\mathrm M5\right)$$

The parameters, α, β, γ and δ each refer to a general intercept, scale, shape, and asymptote (e.g., to ensure estimates to not exceed 1).

Models M6 were fitted as follows:

$$\mathrm{EQ}-5\mathrm D-5{\mathrm L}_{\mathrm{VH}/\mathrm{AL}=}\dots.\mathrm\alpha+{\mathrm\beta}_1\ast\mathrm{VNS}+{\mathrm\beta}_2\ast\mathrm{VNS}^2+{\mathrm\beta}_3\ast\mathrm{VNS}^3+\mathrm\beta^4\ast\mathrm{VNS}^4\left(\mathrm M6\right)$$

$$\mathrm{EQ}-5\mathrm D-5{\mathrm L}_{\mathrm{VH}/\mathrm{AL}=}\dots.\mathrm\alpha+{\mathrm\beta}_1\ast\mathrm{RPS}+{\mathrm\beta}_2\ast\mathrm{RPS}^2+{\mathrm\beta}_3\ast\mathrm{RPS}^3\left(\mathrm M6\right)$$

Since VNS has 5 categories and RPS has 4, a polynomial regression of orders 4 and 3 were fitted to ensure adequate degrees of freedom to estimate all parameters. For RPS, a covariate for vitiligo location was included to compare utility predictions between hands/feet and head/face, this covariate was not statistically significant and not included in the final model however results for the inclusion of this covariate are reported in Supplementary Table 5. The results for the inclusion of age in the RPS polynomial model are also reported in Supplementary Table 5.

#### HRQoL effects and relevance to re-pigmentation

The relationship between responses based on RPS, TVS, EQ-5D and VNS were investigated using model-based estimates. Logistic regression models were used to determine whether any viable cut-off scores for TVS or EQ-5D utilities could be associated with a VNS score of 4 or 5. All analyses were conducted using SAS® v9.4 [31]. Where RPS scores suggested de-pigmentation (i.e., worsening/negative scores), these were taken into account.

## Results

### Demographics and patient disposition

Overall, for EQ-5D, 58% of patients had non missing responses at 9 months; for VitiQoL, VNS and re-pigmentation, these were respectively 50%, 80% and 67% at 9 months (Supplementary Table 1). A total of 517 participants were randomised. The mean (standard deviation (SD)) ages were 39 (20.0), 37 (18.9) and 37 years (19.1) for TCS, NB-UVB and the TCS + NB-UVB combination, respectively; 52% were males; 64% Caucasian. Full details of the trial results are reported elsewhere [1] (Table 1).

**Table 1:** Baseline characteristics

		TCS (*N* = 173)	NB-UVB (*N* = 169)	TCS + NB-UVB (*N* = 175)	Total (*N* = 517)
**Gender**					
Female	n (%)	98 (57%)	81 (48%)	70 (40%)	249 (48%)
Male	n (%)	75 (43%)	88 (52%)	105 (60%)	268 (52%)
**Ethnicity**					
White	n (%)	112 (65%)	114 (67%)	104 (59%)	330 (64%)
Indian	n (%)	13 (8%)	13 (8%)	10 (6%)	36 (7%)
Pakistani	n (%)	12 (7%)	15 (9%)	27 (15%)	54 (10%)
Bangladeshi	n (%)	4 (2%)	4 (2%)	4 (2%)	12 (2%)
Black	n (%)	5 (3%)	3 (2%)	7 (4%)	15 (3%)
Chinese	n (%)	2 (1%)	1 (1%)	1 (1%)	4 (1%)
Other Asian (not Chinese)	n (%)	5 (3%)	6 (4%)	6 (3%)	17 (3%)
Mixed race	n (%)	9 (5%)	6 (4%)	6 (3%)	21 (4%)
Other	n (%)	10 (6%)	7 (4%)	9 (5%)	26 (5%)
Missing	n (%)	1 (1%)	0	1 (1%)	2 (< 1%)
**Age at Randomization (years)**					
	N	173	169	175	517
	Mean (SD)	38.6 (20.0)	36.9 (18.9)	37.0 (19.1)	37.5 (19.3)
**Age of adults at Randomization (years)**					
	N	133	130	135	398
	Mean (SD)	46.7 (15.2)	44.7 (14.0)	44.8 (14.2)	45.4 (14.47)
**Age of children at Randomization (years)**					
	N	40	39	40	119
	Mean (SD)	11.7 (3.7)	10.8 (3.5)	10.6 (3.3)	11.03 (3.5)

*SD *Standard deviation, *TCS *Topical Corticoid Steroids. *NB-UVB *Narrowband Ultraviolet B (a form of phototherapy)

A total of 914 (EQ-5D) observations covering 76 health states were observed at screening, months 9 and 21 (Table 2). The most frequent health state was '11111' (58%); 3 utility scores were < 0 for the VH crosswalk and 4 < 0 for the Alava crosswalk. The observed mean (standard error (SE)) EQ-5D utility score was 0.9067 (0.005) for VH and 0.9008 (0.005) for Alava (Table 2). Correlations (Spearman’s) between the EQ-5D and individual VitiQoL items were low: between − 0.31 (TVS) and − 0.12 (VitiQoL Q13), suggesting utility increase (higher scores) for lower VitiQoL scores. For VNS and RPS, correlations between EQ-5D and VNS, RPS were 0.04 (Supplementary Table 2). The mean utility within each VNS/RPS category was highly correlated with EQ-5D (correlation of 0.879 and 0.818 respectively), suggesting a clear increase in utility as VNS scores and RPS increase (Supplementary Table 2). In general, correlations were similar between VH and Alava crosswalks. The observed correlations suggest suitable mappings are plausible for each of VitiQoL, VNS and RPS and baseline total VitiQoL scores between hands vs. face, showed no statistical differences (*p* = 0.331).

**Table 2:** Summary of health states & EQ-5D-5 L utility scores

*N* = 914 (Number of complete EQ-5D-5 L observations at Screening, Months 9 and 21)	
EQ-5D-5 L # observations	914 Complete observations
Health states (range)	76 (11111, 44453)
Most Frequent HS	11111 (58%)
**VH EQ-5D**: [range] {# of data points < 0}	[1, -0.080] {3 data points < 0}
Observed Mean (SE)	0.9067 (0.005)
**Alava EQ-5D**: [range] {# of data points < 0}	[0.989, -0.134] {4 data points < 0}
Observed Mean (SE)	0.9008 (0.005)

*HS *Number of health states, *VH *Van Hout Crosswalk, *SE *Standard Error

### Mapping model performance

For all models, the performance metrics are reported in Tables 3 and 4. Final model estimates (coefficients) are reported in Supplementary Tables 3a and 3b, and final mapping algorithms are presented in Supplementary Table 4. Results from the Alava crosswalk, in general provided a ‘better’ mapping algorithm:

**Table 3:** Model performance: VitiQoL mapping algorithms

		Van Hout Crosswalk			Alava Crosswalk		
		M1 (Linear)	M2 (Multivariate)	M3 (Bayesian Linear)	M1 (Linear)	M2 (Multivariate)	M3 (Bayesian Linear)
**VitiQoL**	**MAE**	0.102	0.107	0.101	0.022	0.093	0.096
	**RMSE**	0.1507	0.146	0.1506	0.149	0.143	0.149
	**Predicted Mean (SE)**	0.8953 (0.002)	0.8939 (0.002)	0.8951 (0.002)	0.8916 (0.002)	0.8913 (0.002)	0.8912 (0.002)
	**Observed Mean (SE)**	0.9067 (0.005)	0.9067 (0.005)	0.9067 (0.005)	0.9008 (0.005)	0.9008 (0.005)	0.9008 (0.005)
	**Difference (Mean)**	0.0114	0.0128	0.0116	0.0092	0.0095	0.0096
	**AIC/DIC**^**#**^	-1361	-1257	-1377#	-1382	-1319	-1399#
	**Predicted QALY**	1.5728 (0.006)	1.5782 (0.007)	1.5725 (0.006)	1.5660 (0.005)	1.5754 (0.007)	1.5654 (0.006)
	**Observed QALY**	1.5979 (0.017)	1.5979 (0.017)	1.5979 (0.017)	1.5887 (0.017)	1.5887 (0.017)	1.5887 (0.017)
	**Difference (QALY)**	0.0251	0.0197	0.0254	0.0227	0.0133	0.0233

*M1 *Linear Model, *M2 *Linear Multivariate Model, *M3 *Bayesian Linear Model, *MAE *Mean Absolute Error, *RMSE *Root Mean Squared Error, *QALY *Quality Adjusted Life Year, *AIC *Akaike Information Criterion, *SD *Standard Deviation, *SE *Standard Error. Difference (Mean): Observed - Predicted, QALY estimates derived from baseline, month 9 and month 21 data

**Table 4:** Model performance: VNS, RPS mapping algorithms

	Van Hout Crosswalk			Alava Crosswalk		
	**M4 (Linear)**	**M5 (Non-Linear)**	**M6 (Polynomial)**	**M4 (Linear)**	**M5 (Non-Linear)**	**M6 (Polynomial)**
**VNS**						
**MAE**	0.108	0.019	0.0012	0.100	0.118	0.0011
**RMSE**	0.152	0.021	0.00021	0.151	0.016	0.0022
**Predicted Mean (SE)**	0.9115 (0.0003)	0.8851 (0.0005)	0.9081 (0.0003)	0.9000 (0.0002)	0.8996 (0.0003)	0.8939 (0.0003)
**Observed Mean (SE)**	0.9067 (0.005)	0.9067 (0.005)	0.9067 (0.005)	0.9008 (0.005)	0.9008 (0.005)	0.9008 (0.005)
**Difference (Mean)**	-0.0048	0.0216	-0.0014	0.0008	0.0012	0.0069
**AIC**	-681	-691	-2850	-701	-714	-2899
**Predicted QALY**	1.5967 (0.0013)	1.5503 (0.0020)	1.5899 (0.0013)	1.5758 (0.0007)	1.5742 (0.0011)	1.5646 (0.0011)
**Observed QALY**	1.5979 (0.017)	1.5979 (0.017)	1.5979 (0.017)	1.5887 (0.017)	1.5887 (0.017)	1.5887 (0.017)
**Difference (QALY)**	0.0012	0.0476	0.0080	0.0129	0.0145	0.0241
**RPS**						
**MAE**	0.107	0.031	0.001	0.100	0.014	0.0008
**RMSE**	0.153	0.031	0.00018	0.151	0.016	0.0011
**Predicted Mean (SE)**	0.9068 (0.0004)	0.8834 (0.0002)	0.9069 (0.0004)	0.8997 (0.0003)	0.8917 (0.0003)	0.8984 (0.0004)
**Observed Mean (SE)**	0.9067 (0.005)	0.9067 (0.005)	0.9067 (0.005)	0.9008 (0.005)	0.9008 (0.005)	0.9008 (0.005)
**Difference (Mean)**	-0.0001	0.0233	-0.0002	0.0011	0.0091	0.0024
**AIC**	-664	-697	-2743	-683	-698.4	-2888
**Predicted QALY**	1.5862 (0.0014)	1.5449 (0.0007)	1.5859 (0.0013)	1.5739 (0.0012)	1.5594 (0.0011)	1.5699 (0.0017)
**Observed QALY**	1.5979 (0.017)	1.5979 (0.017)	1.5979 (0.017)	1.5887 (0.017)	1.5887 (0.017)	1.5887 (0.017)
**Difference (QALY)**	0.0117	0.053	0.012	0.0148	0.0293	0.0188

*M4 *Linear Model, *M5 *Non-Linear Model, *M6 *Polynomial Model (VNS M6: Polynomial regression of orders 4, *RPS M6 *Polynomial regression of orders 3), Re-pigmentation Categories: 0-24% [25], 25-49% [50], 50-74% [75], 75-100% [100], *MAE *Mean Absolute Error, *RMSE *Root Mean Squared Error, *QALY *Quality Adjusted Life Year, *AIC *Akaike Information Criterion, *SD *Standard Deviation, *SE *Standard Error, *VNS *Vitiligo Noticeability Scale, *RPS *Re-pigmentation Score. Difference (Mean): Observed - Predicted; QALY estimates derived from baseline, month 9 and month 21 data

### Mapping from VitiQoL

**Fig. 1:**
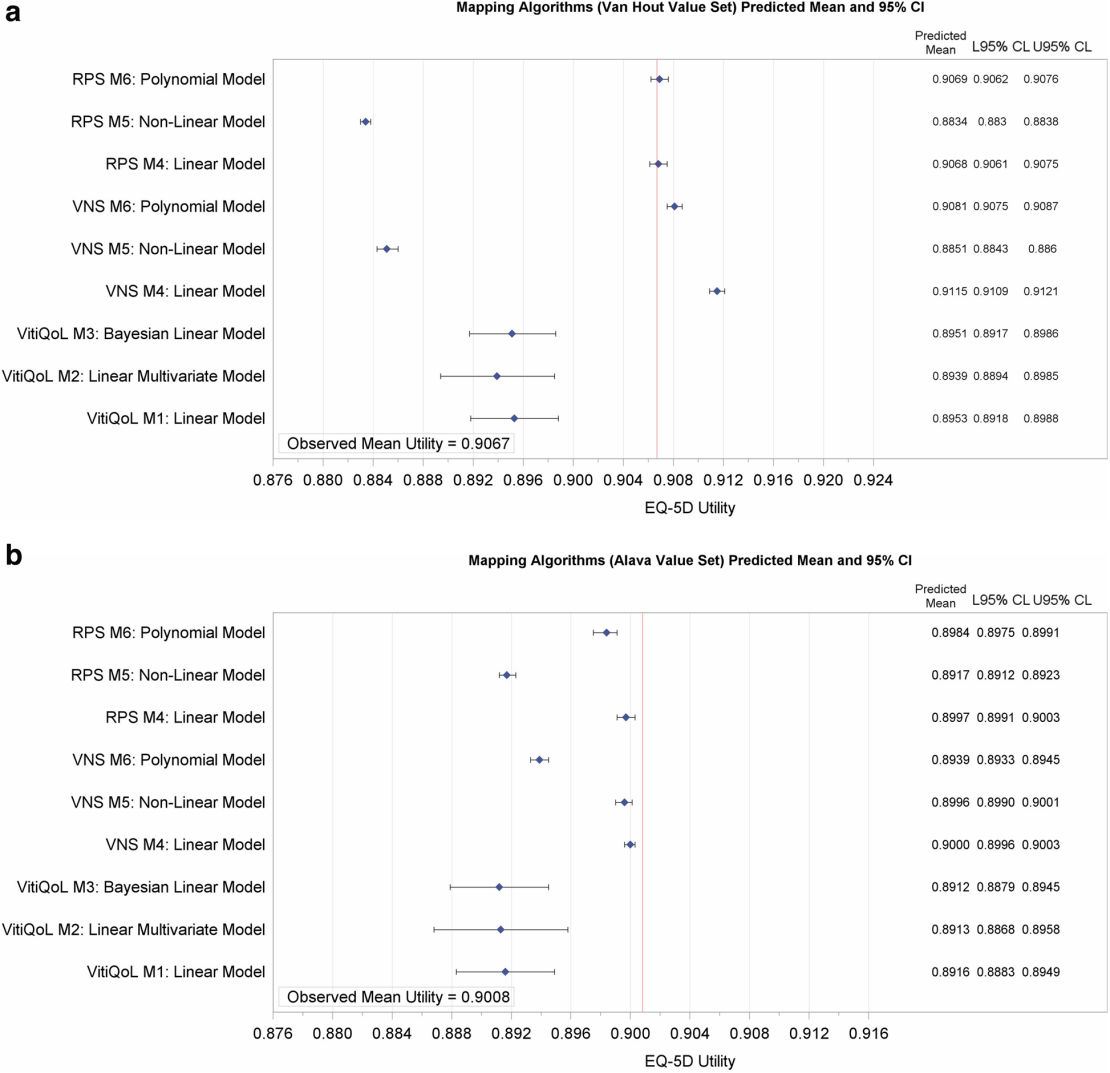
Observed vs Predicted Mean Utilities (Forest Plot). M1: Linear Model; M2: Linear Multivariate Model; M3: Bayesian Linear Model ; M4: Linear Model; M5: Non-Linear Model; M6: Polynomial Model (VNS M6: Polynomial regression of orders 4, RPS M6: Polynomial regression of orders 3); L95% CL: Lower 95% Confidence Level; U95% CL: Upper 95% Confidence Level; VNS: Vitiligo Noticeability Scale; RPS: Re-pigmentation Score

**Fig. 2:**
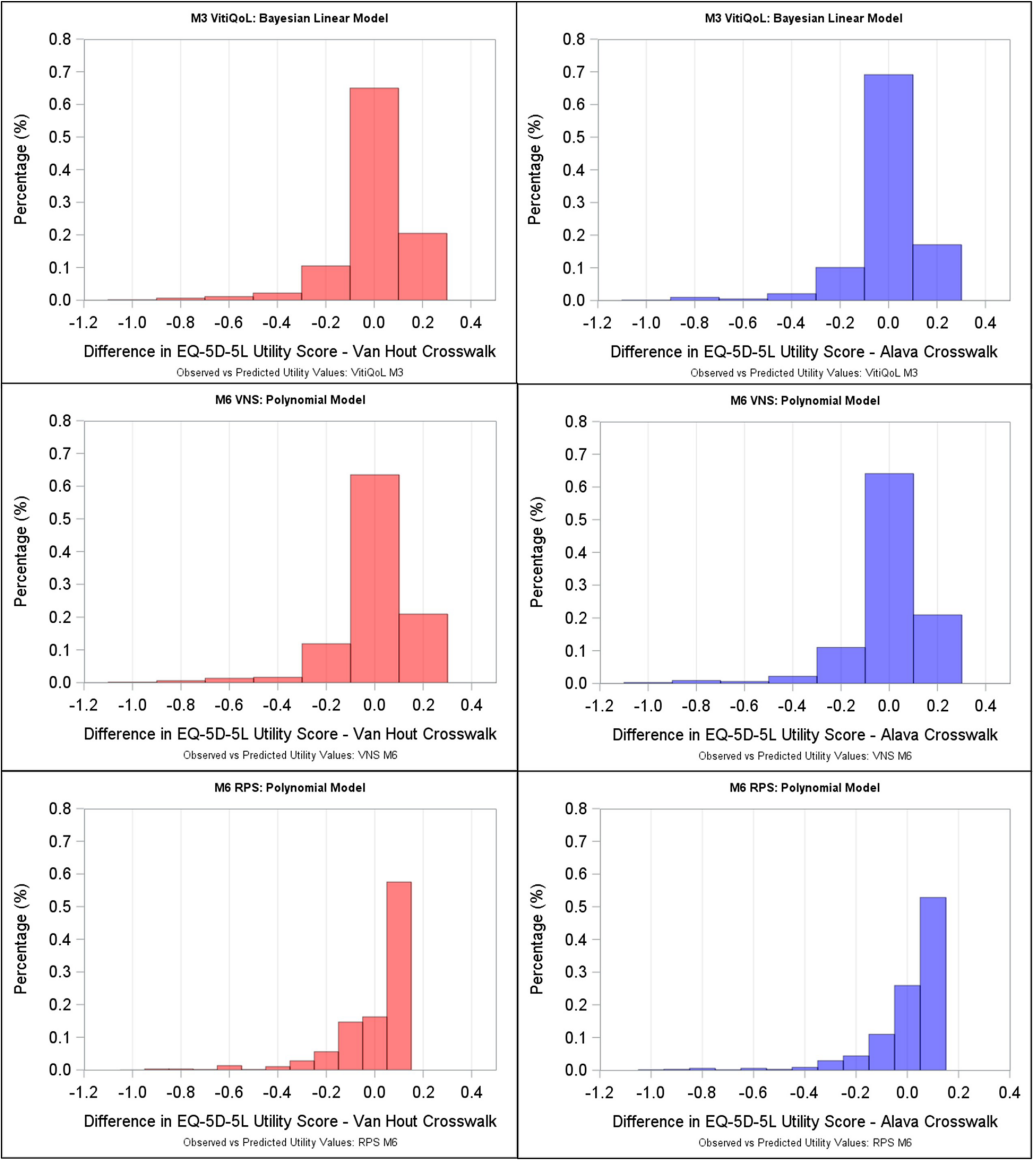
Distribution of Differences in Predicted vs Observed Utility Values. M3: Bayesian Linear Model ; M6: Polynomial Model (VNS M6: Polynomial regression of orders 4, RPS M6: Polynomial regression of orders 3)

The results for VitiQoL were broadly similar between models, however the ‘best’ fitting model was considered to be the BLM (M3) using the TVS (Alava) (Table 3, Figs. 1 and 2): DIC= -1399; observed mean (SE) vs. predicted means were: 0.9008 (0.005) vs 0.8912 (0.002); difference in mean QALY of 0.0233; RMSE of 0.149 and MAE of 0.096. The predicted EQ-5D utility scores based on VitiQoL are therefore best estimated as (Supplementary Table 4):

$$\mathrm{EQ}-5\mathrm D\;{\mathrm{Utility}}_{\mathrm{AL}}^{\mathrm{ViitQoL}}=0.9652-0.00205\ast\mathrm{Total}\;\mathrm{VitiQoL}\;\mathrm{Score}\;\left[\mathrm M3\;\mathrm{Alava}\right]$$

### Mapping from VNS

Results from each of the models for VNS (Table 4, Figs. 1 and 2) show better performance and fit with non-linear models. In particular, the polynomial models (M6) were best. Moreover, the fit at a specific VNS score category was also good (Supplementary Fig. 2): AIC = -2899; observed vs. predicted mean utility was 0.9008 vs. 0.8939; the RMSE was lowest for M6 (RMSE = 0.0022). Figure 2 shows the distribution of differences in observed vs. predicted utilities. The predicted EQ-5D utility scores based on VNS can therefore be estimated as:

$$\mathrm{EQ}-5\mathrm D\;\mathrm{Utility}_{\mathrm{AL}}^{\mathrm{VNS}}=1.1656-0.465\ast\mathrm{VNS}+0.262\ast\mathrm{VNS}^2-0.0599\ast\mathrm{VNS}^3+0.00481\ast\mathrm{VNS}^4\left[\mathrm M6\;\mathrm{Alava}\right]$$

### Mapping models for RPS

For RPS (Table 4, Figs.1 and 2), a slightly better fit was observed with M6, again with the Alava crosswalk: AIC= -2888; observed vs. predicted mean utilities: 0.9008 vs. 0.8984 and in particular, predicted mean utilities at the RPS categories (Supplementary Fig. 2) were closest to the observed for M6: 0.895 vs. 0.891 for RPS 1 (< 25%); 0.924 vs. 0.917 for RPS II (25 − 49%); 0.898 vs. 0.896 for RPS III (50–74%) and 0.936 vs. 0.939 for RPS IV (75 − 100%) for observed vs. predicted mean utilities. Mean QALY differences were also small for M6: 1.5887 vs. 1.5699 for observed vs. predicted QALYs, respectively. Consequently, the predicted EQ-5D utility scores based on RPS can be estimated for VH and Alava (Supplementary Table 4) as:

$$\mathrm{EQ}-5\mathrm D\;\mathrm{Utility}_{\mathrm{AL}}^{\mathrm{RPS}}=0.709+0.0119\ast\mathrm{RPS}-0.000214\ast\mathrm{RPS}^2+0.00000118\ast\mathrm{RPS}^3\;\left[\mathrm M6\;\mathrm{Alava}\right]$$

Although a covariate for Vitiligo location was considered, this was not statistically significant (*p* = 0.420) and had no statistically significant impact on model predictions when included in M6 Alava (mean difference of 0.0004, *p* = 0.420) and was therefore not included in the final model. In general, vitiligo location was not statistically significant in any of the models.

### Testing the final models using independent data (Cross Validation)

The non-linear VNS and RPS models (M6) using out of sample predictions performed best. Model M6 (VNS) yielded mean (SE) observed vs. predicted values of 0.894 (0.007) vs. 0.894 (0.0004); mean difference of 0.0008 (Supplementary Table 6); for RPS, these were 0.901 (0.007) vs. 0.899 (0.0006); mean difference of 0.002. Mean QALYs differences were also broadly similar between Alava and VH (Supplementary Table 6).

### Measures of effect: anchoring VitiQoL, EQ-5D-5 L utilities and VNS to RPS

**Fig. 3:**
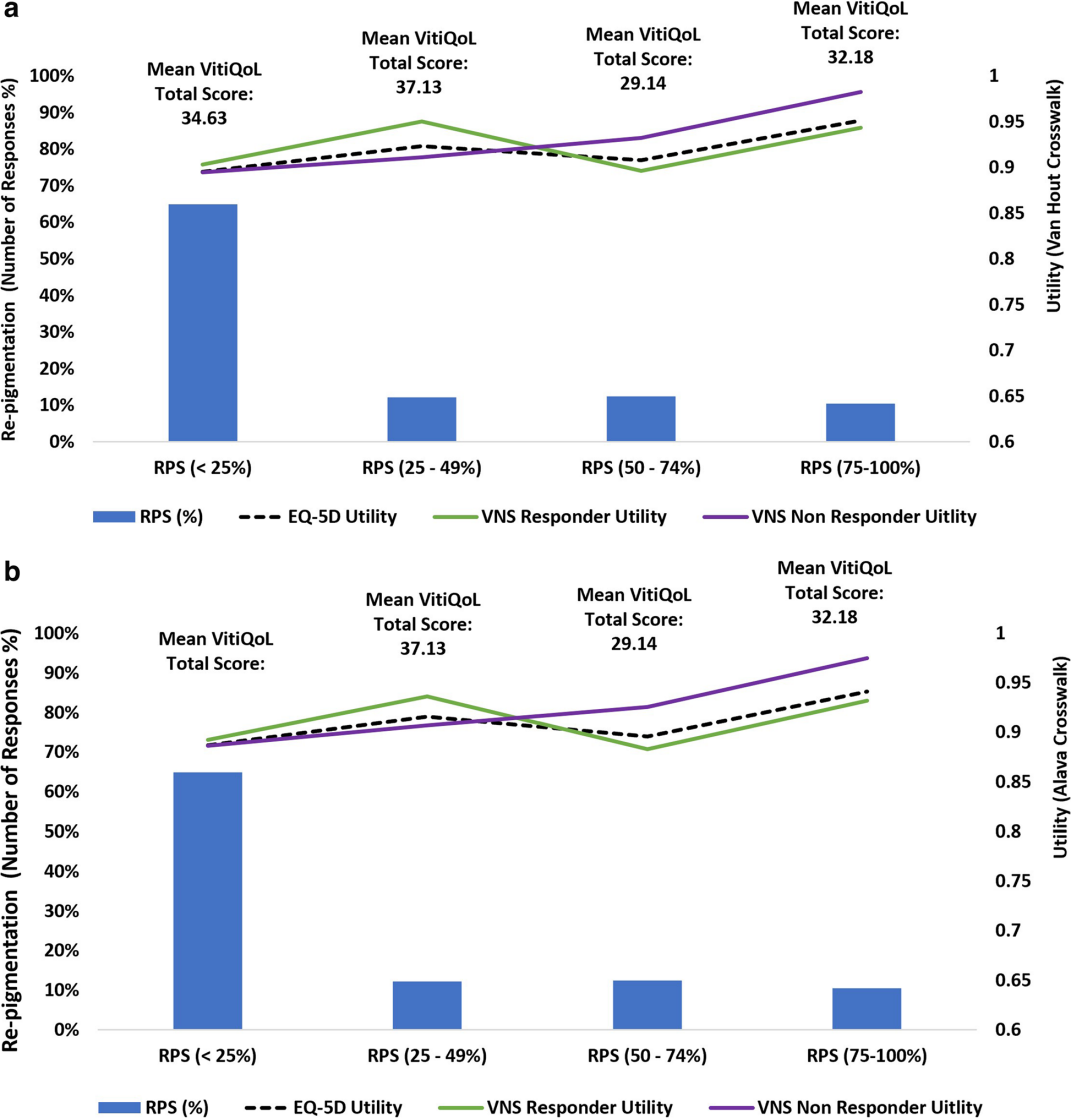
Relationship between VitiQoL Total Score, VNS response, RPS and EQ-5D (Van Hout and Alava Crosswalk) at Month 9. RPS: Re-pigmentation score ; VNS: Vitiligo Noticeability Scale; VNS score 4 or 5: Vitiligo is no longer or a lot less noticeable. VNS responder vs non-responder: VNS Score of 4 or 5 vs VNS Score < 4

**Fig. 4:**
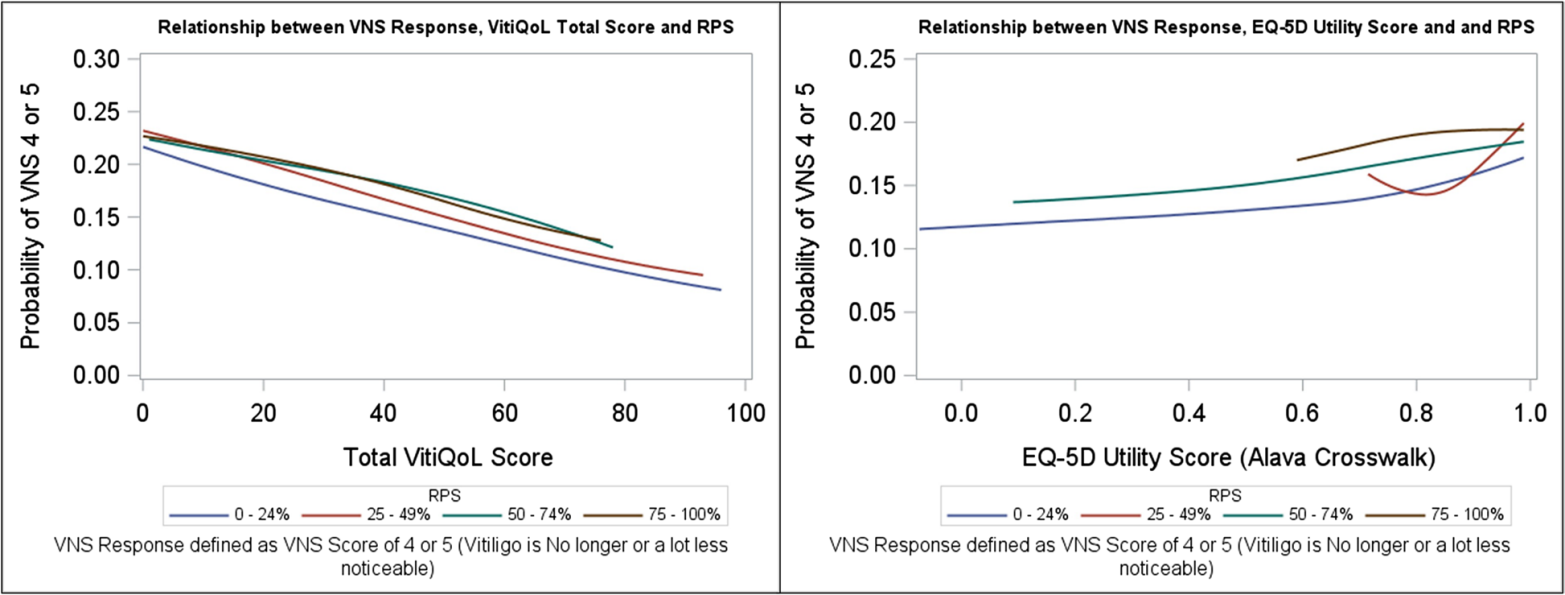
Relationship between VNS Response, VitiQoL Total Score, RPS & EQ-5D Utility Score. RPS: Re-pigmentation score ; VNS: Vitiligo Noticeability Scale; VNS score 4 or 5: Vitiligo is no longer or a lot less noticeable

A relationship between VitiQoL, VNS, RPS and EQ-5D (Table 5; Figs. 3 and 4) is observed: mean TVS decreased (improved HRQoL) as vitiligo became less noticeable (increasing VNS). Assuming VNS scores of 4/5 indicate patients as responders (to treatment), mean scores for VitiQoL amongst VNS responders vs. non responders were 30.18 vs. 35.19, respectively; mean difference of -5.01 [95% CI: -11.09, 1.08; *p* = 0.107].

**Table 5:** Comparison of effect sizes from VitiQoL, VNS, EQ-5D and RPS

	HRQoL Instrument	Response Category	Total VitiQoL	EQ-5D (Van Hout)	EQ-5D (Alava)
			Mean (SD)	Mean (SD)	Mean (SD)
**9 Months**	VNS	1: More Noticeable	37.22 (23.22)	0.900 (0.145)	0.899 (0.124)
		2: As Noticeable	34.61 (22.28)	0.893 (0.168)	0.886 (0.170)
		3: Slightly Less Noticeable	34.43 (23.22)	0.909 (0.154)	0.898 (0.164)
		4: A lot Less Noticeable	30.26 (20.89)	0.917 (0.131)	0.905 (0.145)
		5: No Longer Noticeable	29.73 (24.76)	0.951 (0.108)	0.937 (0.114)
		Responder^#^	30.18 (21.36)	0.922 (0.127)	0.910 (0.140)
		Non-Responder	35.18 (22.80)	0.901 (0.157)	0.893 (0.157)
		Difference^a^ (95% CI; *p*-value)	-5.01 (-11.09, 1.08) *p* = 0.107	0.022 (-0.016, -0.012) *p* = 0.258	0.017 (-0.022, 0.055) *p* = 0.394
**Baseline**	RPS (%)	< 25	35.54 (24.28)	0.896 (0.174)	0.894 (0.166)
		25 to 49	38.35 (22.67)	0.945 (0.071)	0.943 (0.058)
		50–74	34.90 (17.18)	0.914 (0.108)	0.900 (0.111)
		75–100	32.00 (28.67)	0.921 (0.126)	0.915 (0.125)
**9 Months**	RPS (%)	< 25	34.63 (22.62)	0.895 (0.167)	0.887 (0.168)
		25 to 49	37.13 (24.82)	0.923 (0.084)	0.916 (0.080)
		50–74	29.14 (19.83)	0.908 (0.141)	0.896 (0.159)
		75–100	32.18 (22.06)	0.951 (0.103)	0.941 (0.104)
		Responder (≥ 75)	30.45 (20.71)	0.927 (0.127)	0.916 (0.139)
		Non-Responder	35.05 (22.97)	0.899 (0.157)	0.891 (0.157)
		Difference^a^ (95% CI; *p*-value)	-4.60 (-10.79, 1.59) *p* = 0.145	0.027 (-0.011, 0.065) *p* = 0.157	0.024 (-0.014, 0.063) *p* = 0.217

*RPS *Re-pigmentation score, *VNS *Vitiligo Noticeability Scale, *SE *Standard Error; ^#^VNS score 4 or 5: Vitiligo is no longer or a lot less noticeable; ^a^VNS responder vs. non-responder: VNS Score of 4 or 5 vs. VNS Score < 4

 Figure 4 shows the chance of vitiligo noticeability in terms of TVS and utility for varying RPS responses. For patients with the least re-pigmentation (RPS < 25%), a TVS of about 20 points gives around an 18% chance of vitiligo being no longer/a lot less noticeable; for patients with higher re-pigmentation (RPS ≥ 75%), an EQ-5D utility of around 0.80 provides around 19% chance of vitiligo being no longer or a lot less noticeable. For RPS responders (≥ 75% RPS), the mean Total VitiQoL scores were 30.45 vs. 35.05, mean difference of -4.60 [95% CI: -10.79, 1.59; *p* = 0.145] (Table 5). These results suggest a mean difference of 4 or 5 points on TVS yields commensurate benefits on the VNS and RPS scales and potentially discriminate between those with HRQoL improvements and those without. The mean observed vs. predicted EQ-5D utility in the VNS response categories were 0.907 vs. 0.908 for a VNS score 1; 0.888 vs. 0.881 for VNS score 2; 0.903 vs. 0.900 for VNS score 3; 0.905 vs. 0.895 for VNS score 4; 0.932 vs. 0.909 for VNS score 5 (Supplementary Figure 2).

## Discussion

We have provided a way of estimating EQ-5D utilities from two vitiligo specific HRQoL instruments and shown a relationship between CSMs of HRQoL and clinical outcomes such that plausible clinical cut-off scores for RPS can be associated with HRQoL. Several useful mapping algorithms with adequate performance characteristics when compared to available mapping algorithms [18, 19, 21] in literature have been presented. The performance of these vitiligo specific algorithms appear more favourable compared to those reported in published DLQI algorithms [18, 21]: for example, MAEs for DLQI ranged between 0.1873 and 0.2009 [18], somewhat higher that those reported in this analysis. We have also for the first time compared the algorithms between two crosswalks: VH and Alava and showed these to provide broadly similar results. In addition, we demonstrated a coherent relationship between VitiQoL, VNS, RPS and VNS which may be useful for designing future research in vitiligo; and finally, we offer an approach that could provide a way of relating HRQoL benefits in vitiligo with clinical effects that can yield tangible cut-off scores. It is important to note, appropriate adjustments to RPS classification may be required before implementation of the RPS mapping algorithms, depending on the type of re-pigmentation response recorded. For example, if de-pigmentation occurs, this can be incorporated by creating extra category/categories reflecting a worsening condition.

There are several limitations to this research. Firstly, all published mapping algorithms when applied to independent data will reflect differences in factors such as patient characteristics, trial conditions and assessment points. Secondly, by 9 months only around 60% of the data in the HI-Light trial were available for the mapping model and the data did not include a complete range of EQ-5D-5 L health states; thirdly, scales such as the VNS and RPS are discrete and as such patient level modelling tends to classify predictions into a limited number of possibilities. Fourthly, in an attempt to anchor clinical outcomes with HRQoL, the chance of no or little noticeability did not exceed much more than 20%, despite high HRQoL benefits in measures such as EQ-5D and Total VitiQoL score. Finally, there is a concern that a mapped utility has no known immediate health state (a type of double mapping): for example, a utility value of 0.193 has a health state of '11153', whereas a predicted (mapped) utility of 0.866 has no known health state profile (UK tariff). However, this is also true for any mean utility computed from known health states and therefore would not seem to be a valid objection. Despite these limitations, the mapping algorithms presented are a ‘first’ in vitiligo that compares two different crosswalks with performance metrics similar or better to that reported in mapping literature. In addition, we provide a basis for further research in delineating cut-off scores that can anchor clinical outcomes such as RPS with HRQoL.

## Conclusion

We have shown that mapping EQ-5D with each of VitiQoL, VNS and clinical measures such as RPS is plausible. Mapping with VNS and RPS appears to show superior performance than with VitiQoL and a relationship between EQ-5D-5 L utility, VNS, VitiQoL and RPS can be considered as a basis for defining clinically meaningful HRQoL differences.

### Supplementary Information

**Additional file 1: Supplementary Figure 1. **Observed vs Predicted QALY Estimates (Forest Plot). M1: Linear Model; M2: Linear Multivariate Model; M3: Bayesian Linear Model ; M4: Linear Model; M5: Non-Linear Model; M6: Polynomial Model (VNS M6: Polynomial regression of orders 4, RPS M6: Polynomial regression of orders 3); QALY: Quality Adjusted Life Year; L95% CL: Lower 95% Confidence Level; U95% CL: Upper 95% Confidence Level; VNS: Vitiligo Noticeability Scale; RPS: Re-pigmentation Score; QALY estimates derived from baseline, month 9 and month 21 data.

**Additional file 2: Supplementary Figure 2.** Mean Utility Scores by VNS & RPS. M6: Polynomial Model (VNS M6: Polynomial regression of orders 4, RPS M6: Polynomial regression of orders 3). VNS: Vitiligo Noticeability Scale; RPS: Re-pigmentation Score.

**Additional file 3:** **Supplementary Table 1. **Data Completeness. RPS: Re-pigmentation score ; VNS: Vitiligo Noticeability Scale.

**Additional file 4: Supplementary Table 2.** Correlation between EQ-5D, VitiQoL, VNS and RPS. RPS: Re-pigmentation score ; VNS: Vitiligo Noticeability Scale; VNS score 4 or 5: Vitiligo is no longer or a lot less noticeable; r_s_: Spearman’s rank correlation.

**Additional file 5: Supplementary Table 3a.** Model Parameter Estimates – VitiQoL Mapping Algorithms. M1: Linear Model; M2: Linear Multivariate Model; M3: Bayesian Linear Model; SE: Standard; *statistically significant at 2 sided 5% level or posterior probability of rejecting Null hypothesis (slope=0) is >97.5%.

**Additional file 6: Supplementary Table 3b.** Model Parameter Estimates – VNS/RPS Mapping Algorithms. M4: Linear Model; M5: Non-Linear Model; M6: Polynomial Model (VNS M6: Polynomial regression of orders 4, RPS M6: Polynomial regression of orders 3); SE: Standard Error; *statistically significant at 2 sided 5% level or posterior probability of rejecting Null hypothesis (slope=0) is >97.5%.

**Additional file 7: Supplementary Table 4.** List of Mapping Algorithms: Van Hout and Alava Crosswalks. M1: Linear Model; M2: Linear Multivariate Model; M3: Bayesian Linear Model ; M4: Linear Model; M5: Non-Linear Model; M6: Polynomial Model (VNS M6: Polynomial regression of orders 4, RPS M6: Polynomial regression of orders 3). TVS : Total VitiQoL Score; items 1 to 16 are the scores from the VitiQoL questions 1 to 16 ; ^#^MV Linear: Multivariate linear for VitiQoL for all 16 items; VNS: Vitiligo Noticeability Scale; RPS: Re-pigmentation; Re-pigmentation Categories: 0-24% [25], 25-49% [50], 50-74% [75], 75-100% [100].

**Additional file 8: Supplementary Table 5.** Model Parameter Estimates – RPS Alava Mapping Algorithm (inclusion of Vitiligo Area/Age). RPS M7/M8: Polynomial regression of orders 3; SE: Standard Error; *statistically significant at 2 sided 5% level or posterior probability of rejecting Null hypothesis (slope=0) is >97.5%.

**Additional file 9: Supplementary Table 6.** Cross Validation Results (VNS, RPS) – Final Models. M6: Polynomial Model (VNS M6: Polynomial regression of orders 4, RPS M6: Polynomial regression of orders 3). Observed and predicted means generated from randomly selected datasets for cross validation purposes, two separate datasets were used for the VNS M6 predictions and RPS M6 predictions. Difference: Observed – Predicted. QALY: Quality Adjusted Life Year; SE: Standard Error; VNS: Vitiligo Noticeability Scale; RPS: Re-pigmentation Score; QALY estimates derived from baseline, month 9 and month 21 data.

## Data Availability

The data that support the findings of this study were provided by the University of Nottingham CTU, however restrictions apply to the availability of these data, which were used under agreement for the research carried out, and so are not publicly available.

## Abbreviations

HRQoL: Health Related Quality of Life
VitiQoL: Vitiligo specific Quality of Life Instrument
VNS: Vitiligo Noticeability Scale
RPS: Re-Pigmentation Scores
VASI: Vitiligo Area Scoring Index
QALY: Quality Adjusted Life Years
CSM: Condition Specific Measures
HTA: Health Technology Assessment
EADV: European Academy of Dermatology and Venerology
VIS: Vitiligo Impact Scale
DLQI: Dermatology Life Quality Index
NICE: National Institute for Health and Care Excellence
DSU: Decision Support Unit
RCT: Randomized Controlled Trial
MAE: Mean Absolute Error
RMSE: Root Mean Square Error
AIC: Akaike information criterion
BIC: Bayesian information criterion
DIC: Deviance information criterion
SE: Standard Error
SD: Standard Deviation
