# Supplementary material for: Estimating health related quality of life effects in vitiligo. Mapping EQ-5D-5 L utilities from vitiligo specific scales: VNS, VitiQoL and re-pigmentation measures using data from the HI-Light trial
Source: Health Qual Life Outcomes. 2023 Aug 10;21:85. doi: 10.1186/s12955-023-02172-4 (PMC10413598; doi:10.1186/s12955-023-02172-4)
Supplement: Supplementary file 1 — Additional file 1: Supplementary Figure 1. Observed vs Predicted QALY Estimates (Forest Plot). M1: Linear Model; M2: Linear Multivariate Model; M3: Bayesian Linear Model ; M4: Linear Model; M5: Non-Linear Model; M6: Polynomial Model (VNS M6: Polynomial regression of orders 4, RPS M6: Polynomial regression of orders 3); QALY: Quality Adjusted Life Year; L95% CL: Lower 95% Confidence Level; U95% CL: Upper 95% Confidence Level; VNS: Vitiligo Noticeability Scale; RPS: Re-pigmentation Score; QALY estimates derived from baseline, month 9 and month 21 data. [file 12955_2023_2172_MOESM1_ESM.docx]

**Supplementary Table 1: Data Completeness**

|  | **Baseline**  **(N=517)** | **9 months**  **(N=517)** | **21 Months**  **(N=517)** |
| --- | --- | --- | --- |
| **EQ-5D-5L** | 435 (84%) | 299 (58%) | 180 (35%) |
| **VitiQoL** |  |  |  |
| VitiQol Q1 | 389 (75%) | 257 (50%) | 176 (34%) |
| VitiQol Q2 | 389 (75%) | 257 (50%) | 177 (34%) |
| VitiQol Q3 | 388 (75%) | 257 (50%) | 175 |
| VitiQol Q4 | 390 (75%) | 257 (50%) | 176 (34%) |
| VitiQol Q5 | 390 (75%) | 256 (50%) | 176 (34%) |
| VitiQol Q6 | 390 (75%) | 256 (50%) | 175 (34%) |
| VitiQol Q7 | 389 (75%) | 257 (50%) | 176 (34%) |
| VitiQol Q8 | 389 (75%) | 257 (50%) | 176 (34%) |
| VitiQol Q9 | 390 (75%) | 257 (50%) | 177 (34%) |
| VitiQol Q10 | 390 (75%) | 257 (50%) | 177 (34%) |
| VitiQol Q11 | 389 (75%) | 255 (49%) | 177 (34%) |
| VitiQol Q12 | 390 (75%) | 256 (50%) | 177 (34%) |
| VitiQol Q13 | 390 (75%) | 256 (50%) | 177 (34%) |
| VitiQol Q14 | 390 (75%) | 256 (50%) | 176 (34%) |
| VitiQol Q15 | 389 (75%) | 254 (49%) | 176 (34%) |
| VitiQoL Q16 | 382 (74%) | 250 (48%) | 173 (34%) |
| **Total VitiQoL Score** | 390 (75%) | 257 (50%) | 177 (34%) |
| **VNS** | 232 (45%) | 416 (80%) | 100 (19%) |
| **RPS** | 224 (43%) | 345 (67%) | 100 (19%) |

RPS: Re-pigmentation score ; VNS: Vitiligo Noticeability Scale.
