# Supplementary material for: Estimating health related quality of life effects in vitiligo. Mapping EQ-5D-5 L utilities from vitiligo specific scales: VNS, VitiQoL and re-pigmentation measures using data from the HI-Light trial
Source: Health Qual Life Outcomes. 2023 Aug 10;21:85. doi: 10.1186/s12955-023-02172-4 (PMC10413598; doi:10.1186/s12955-023-02172-4)
Supplement: Supplementary file 2 — Additional file 2: Supplementary Figure 2. Mean Utility Scores by VNS & RPS. M6: Polynomial Model (VNS M6: Polynomial regression of orders 4, RPS M6: Polynomial regression of orders 3). VNS: Vitiligo Noticeability Scale; RPS: Re-pigmentation Score. [file 12955_2023_2172_MOESM2_ESM.docx]

**Supplementary Table 2: Correlation between EQ-5D, VitiQoL, VNS and RPS**

| Parameter | EQ-5D VH | EQ-5D Alava | VNS | RPS |
| --- | --- | --- | --- | --- |
|  | r_s_; p-value | r_s_; p-value | r_s_; p-value | r_s_; p-value |
|  |  |  |  |  |
| VitiQoL Q1 | -0.22466 p<0.0001 | -0.30276 p<0.0001 | -0.04065 p=0.3198 | 0.02162 p=0.6106 |
| VitiQoL Q2 | -0.25443 p<00.001 | -0.32751 p<0.0001 | -0.04388 p=0.2829 | 0.01545 p=0.7160 |
| VitiQoL Q3 | -0.29301 p<0.0001 | -0.33590 p<0.0001 | -0.02651 p=0.5176 | -0.00069 p=0.9872 |
| VitiQoL Q4 | -0.26563 p<0.0001 | -0.29307 p<0.0001 | -0.00960 p=0.8145 | -0.03114 p=0.4636 |
| VitiQoL Q5 | -0.22321 p<0.001 | -0.29547 p<0.0001 | -0.03931 p=0.3373 | -0.05450 p=0.1999 |
| VitiQoL Q6 | -0.25358 p<0.0001 | -0.31776 p<0.0001 | -0.01949 p=0.6341 | -0.05944 p=0.1620 |
| VitiQoL Q7 | -0.23718 p<0.0001 | -0.31828 p<0.0001 | -0.03075 p=0.4525 | -0.03854 p=0.3649 |
| VitiQoL Q8 | -0.18075 p<0.0001 | -0.25653 p<0.0001 | -0.12649 p=0.0019 | -0.13234 p=0.0018 |
| VitiQoL Q9 | -0.27102 p<0.0001 | -0.31829 p<0.0001 | -0.05547 p=0.1744 | -0.06196 p=0.1442 |
| VitiQoL Q10 | -0.32078 p<0.0001 | -0.39034 p<0.0001 | -0.04840 p=0.2361 | -0.05772 p=0.1737 |
| VitiQoL Q11 | -0.28288 p<0.0001 | -0.31249 p<0.0001 | -0.05022 p=0.2205 | -0.07872 p=0.0638 |
| VitiQoL Q12 | -0.22233 p<0.0001 | -0.31012 p<0.0001 | -0.04191 p=0.3058 | 0.02984 p=0.4826 |
| VitiQoL Q13 | -0.12116 p=0.0005 | -0.13587 p<0.0001 | -0.08510 p=0.0373 | -0.07840 p=0.0647 |
| VitiQoL Q14 | -0.30929 p<0.0001 | -0.30916 p<0.0001 | -0.01680 p=0.6816 | 0.00753 p=0.8594 |
| VitiQoL Q15 | -0.18875 p<0.0001 | -0.25381 p<0.0001 | -0.01598 p=0.6972 | 0.00962 p=0.8215 |
| VitiQoL Q16 | -0.19416 p<0.0001 | -0.25420 p<0.0001 | -0.09854 p=0.0169 | -0.04058 p=0.3440 |
| Total VitiQoL Score | -0.31288 p<0.0001 | -0.39221 p<0.0001 | -0.05112 p=0.2108 | -0.03657 p=0.3890 |
| VNS | 0.04066 p=0.2890 | 0.04939 p=0.1976 | N/A | 0.63860 p<0.0001 |
| RPS | 0.04073 p=0.3105 | 0.04858 p=0.2263 | 0.63860 p<0.0001 | N/A |
| Mean VNS | 0.879 p=0.049 | 0.786 p=0.115 |  |  |
| Mean RPS | 0.818 p=0.182 | 0.771 p=0.229 |  |  |
|  |  |  |  |  |

RPS: Re-pigmentation score ; VNS: Vitiligo Noticeability Scale; VNS score 4 or 5: Vitiligo is no longer or a lot less noticeable; r_s_: Spearman’s rank correlation.
