# Supplementary material for: Estimating health related quality of life effects in vitiligo. Mapping EQ-5D-5 L utilities from vitiligo specific scales: VNS, VitiQoL and re-pigmentation measures using data from the HI-Light trial
Source: Health Qual Life Outcomes. 2023 Aug 10;21:85. doi: 10.1186/s12955-023-02172-4 (PMC10413598; doi:10.1186/s12955-023-02172-4)
Supplement: Supplementary file 3 — Additional file 3: Supplementary Table 1. Data Completeness. RPS: Re-pigmentation score ; VNS: Vitiligo Noticeability Scale. [file 12955_2023_2172_MOESM3_ESM.docx]

**Supplementary Table 3a: Model Parameter Estimates – VitiQoL Mapping Algorithms**

|  | **VH** | | | **Alava** | | |
| --- | --- | --- | --- | --- | --- | --- |
|  | **M1 (Linear)** | **M2 (Linear)** | **M3 (Bayes Linear)** | **M1 (Linear)** | **M2 (Linear)** | **M3 (Bayes Linear)** |
| **Model Parameters** | Estimate (SE) | Estimate (SE) | Estimate (SE) | Estimate (SE) | Estimate (SE) | Estimate (SE) |
| **Intercept Term** | 0.9732 | 0.9291 | 0.9734 | 0.9652 | 0.9178 | 0.9652 |
| **Total VitiQoL Score** | -0.00216 (0.000169) | N/A | -0.00217 (0.000138) | -0.00204 (0.000168) | N/A | -0.00205 (0.000166) |
| **VitiQol Q1** | N/A | 0.000577 (0.00421) | N/A | N/A | 0.000083 (0.00409) | N/A |
| **VitiQol Q2** | N/A | -0.00341 (0.00418) | N/A | N/A | -0.00150 (0.00395) | N/A |
| **VitiQol Q3** | N/A | 0.004364 (0.00372) | N/A | N/A | 0.001952 (0.00356) | N/A |
| **VitiQol Q4** | N/A | -0.00537 (0.00341) | N/A | N/A | -0.00229 (0.00337) | N/A |
| **VitiQol Q5** | N/A | 0.003876 (0.00386) | N/A | N/A | 0.001994 (0.00371) | N/A |
| **VitiQol Q6** | N/A | 0.002001 (0.00387) | N/A | N/A | 0.002850 (0.00367) | N/A |
| **VitiQol Q7** | N/A | -0.00115 (0.00431) | N/A | N/A | -0.00013 (0.00418) | N/A |
| **VitiQol Q8** | N/A | 0.005252 (0.00249) | N/A | N/A | 0.005247 (0.00241)* | N/A |
| **VitiQol Q9** | N/A | -0.00133 (0.00369) | N/A | N/A | -0.00301 (0.00361) | N/A |
| **VitiQol Q10** | N/A | -0.01777 (0.00427)* | N/A | N/A | -0.01468 (0.00412)* | N/A |
| **VitiQol Q11** | N/A | -0.01195 (0.00409)* | N/A | N/A | -0.01360 (0.00385)* | N/A |
| **VitiQol Q12** | N/A | 0.002250 (0.00226) | N/A | N/A | 0.000708 (0.00216) | N/A |
| **VitiQol Q13** | N/A | 0.000860 (0.00205) | N/A | N/A | 0.004218 (0.00209) | N/A |
| **VitiQol Q14** | N/A | -0.02216 (0.00399)* | N/A | N/A | -0.02220 (0.00382)* | N/A |
| **VitiQol Q15** | N/A | -0.00004 (0.00269) | N/A | N/A | 0.001753 (0.00263) | N/A |
| **VitiQoL Q16** | N/A | 0.003234 (0.00381) | N/A | N/A | -0.00230 (0.0036) | N/A |

M1: Linear Model; M2: Linear Multivariate Model; M3: Bayesian Linear Model; SE: Standard; *statistically significant at 2 sided 5% level or posterior probability of rejecting Null hypothesis (slope=0) is >97.5%
