# Supplementary material for: Estimating health related quality of life effects in vitiligo. Mapping EQ-5D-5 L utilities from vitiligo specific scales: VNS, VitiQoL and re-pigmentation measures using data from the HI-Light trial
Source: Health Qual Life Outcomes. 2023 Aug 10;21:85. doi: 10.1186/s12955-023-02172-4 (PMC10413598; doi:10.1186/s12955-023-02172-4)
Supplement: Supplementary file 4 — Additional file 4: Supplementary Table 2. Correlation between EQ-5D, VitiQoL, VNS and RPS. RPS: Re-pigmentation score ; VNS: Vitiligo Noticeability Scale; VNS score 4 or 5: Vitiligo is no longer or a lot less noticeable; rs: Spearman’s rank correlation. [file 12955_2023_2172_MOESM4_ESM.docx]

**Supplementary Table 3b: Model Parameter Estimates – VNS/RPS Mapping Algorithms**

|  | **Model** | **VH** | **Alava** |
| --- | --- | --- | --- |
|  |  | Estimate (SE) | Estimate (SE) |
| **VNS Models** | **M4 (Linear)** |  |  |
|  | Intercept Term | 0.8851 | 0.8857 |
|  | VNS | 0.0104 (0.00568) | 0.00562 (0.00560) |
|  | **M5 (Non-Linear)** |  |  |
|  | **a** | 0.907 (0.0234) | 0.9094 |
|  | **b** | 0.052 (0.042) | 0.0435 (0.0443) |
|  | **g** | -0.129 (0.008) | -0.1139 (0.0065) |
|  | **d** | 0.91 (2.567) | 0.5097 (2.233) |
|  | **M6 (Polynomial)** |  |  |
|  | Intercept Term | 1.0454 | 1.1656 |
|  | VNS | -0.2478 (0.1748) | -0.4652 (0.3178) |
|  | VNS^2^ | 0.1386 (0.1988) | 0.2628 (0.1957) |
|  | VNS^3^ | -0.0312 (0.0496) | -0.0599 (0.0488) |
|  | VNS^4^ | 0.00251 (0.0043) | 0.00481 (0.00424) |
|  |  |  |  |
| **RPS Models** | **M4 (Linear)** |  |  |
|  | Intercept Term | 0.8862 | 0.882 |
|  | RPS | 0.000536 (0.00025)* | 0.000461 (0.00025)* |
|  | **M5 (Non-Linear)** |  |  |
|  | **a** | 4.6859 (15.235) | 9.502 (22.231) |
|  | **b** | 0.00334 (0.0834) | 0.00466 (0.0754) |
|  | **g** | -0.729 (0.962) | -1.5096 (0.881) |
|  | **d** | 182.4 (65.213) | 300.1 (120.34) |
|  | **M6 (Polynomial)** |  |  |
|  | Intercept Term | 0.843 | 0.709 |
|  | RPS | 0.00354 (0.00883)* | 0.0119 (0.00871)* |
|  | RPS^2^ | -0.0000586 (0.000161) | -0.0002147 (0.0001) |
|  | RPS^3^ | 0.000000332 (<0.0001) | 0.00000118 (<0.001) |

M4: Linear Model; M5: Non-Linear Model; M6: Polynomial Model (VNS M6: Polynomial regression of orders 4, RPS M6: Polynomial regression of orders 3); SE: Standard Error; *statistically significant at 2 sided 5% level or posterior probability of rejecting Null hypothesis (slope=0) is >97.5%
