# Supplementary material for: Estimating health related quality of life effects in vitiligo. Mapping EQ-5D-5 L utilities from vitiligo specific scales: VNS, VitiQoL and re-pigmentation measures using data from the HI-Light trial
Source: Health Qual Life Outcomes. 2023 Aug 10;21:85. doi: 10.1186/s12955-023-02172-4 (PMC10413598; doi:10.1186/s12955-023-02172-4)
Supplement: Supplementary file 5 — Additional file 5: Supplementary Table 3a. Model Parameter Estimates – VitiQoL Mapping Algorithms. M1: Linear Model; M2: Linear Multivariate Model; M3: Bayesian Linear Model; SE: Standard; *statistically significant at 2 sided 5% level or posterior probability of rejecting Null hypothesis (slope=0) is >97.5%. [file 12955_2023_2172_MOESM5_ESM.docx]

**Supplementary Table 4: List of Mapping Algorithms: Van Hout and Alava Crosswalks**

| **Mapping**  **Source** | **Model** | **Van Hout** | **Alava** |
| --- | --- | --- | --- |
| **VitiQoL** | **M1: Linear** | 0.9732 -0.00216*TVS | 0.9652 -0.00204*TVS |
|  | **M2: MV Linear** | 0.9291 + 0.000577*v1 -0.00341*v2+0.004364*v3 -0.00537*v4 +0.003876*v5 + 0.002001*v6 -0.00115*v7 + 0.005252*v8 -0.00133*v9 -0.01777*v10 -0.01195*v11 + 0.002250*v12 +0.000860*v13 -0.02216*v14 -0.00004*v15 + 0.003234*v16 | 0.9178 + 0.000083*v1 -0.001501*v2+0.001952*v3 -0.00229*v4 +0.001994*v5 + 0.002850*v6 -0.00013*v7 + 0.005247*v8 -0.00301*v9 -0.01468*v10 -0.01360*v11 + 0.000708*v12 +0.004218*v13 -0.02220*v14 +0.001753*v15 - 0.00230*v16 |
|  | **M3: BLM** | 0.9734-0.00217*TVS | 0.9652-0.00205*TVS |
|  |  |  |  |
| **VNS** | **M4: Linear** | 0.8851 + 0.0104*VNS; | 0.8857 + 0.00562*VNS; |
|  | **M5: Non-Linear** | 0.907 +0.052*VNS - 0.129*log(VNS+0.91) | 0.9094 +0.0435*VNS - 0.1139*log(VNS+0.5097) |
|  | **M6: Polynomial** | 1.0454 - 0.247*VNS+0.1386*VNS^2^ -0.0312*VNS^3^+0.00251*VNS^4^ | 1.1656 - 0.465*VNS+0.262*VNS^2^ -0.0599*VNS^3^+0.00481*VNS^4^ |
|  |  |  |  |
| **RPS** | **M4: Linear** | 0.8862 - 0.000536*RPS | 0.882 - 0.000461*RPS |
|  | **M5: Non-Linear** | 4.6859 +0.00334*RPS- 0.729*log(RPS+182.4) | 9.502+0.00466*RPS – 1.5096*log(RPS+300.1) |
|  | **M6: Polynomial** | 0.843 + 0.00354*RPS-0.0000586*RPS^2^ +0.000000332*RPS^3^ | 0.709 + 0.0119*RPS-0.000214*RPS^2^ +0.00000118*RPS^3^ |

M1: Linear Model; M2: Linear Multivariate Model; M3: Bayesian Linear Model ; M4: Linear Model; M5: Non-Linear Model; M6: Polynomial Model (VNS M6: Polynomial regression of orders 4, RPS M6: Polynomial regression of orders 3). TVS : Total VitiQoL Score; items 1 to 16 are the scores from the VitiQoL questions 1 to 16 ; ^#^MV Linear: Multivariate linear for VitiQoL for all 16 items; VNS: Vitiligo Noticeability Scale ; RPS: Re-pigmentation.
