# Supplementary material for: Estimating health related quality of life effects in vitiligo. Mapping EQ-5D-5 L utilities from vitiligo specific scales: VNS, VitiQoL and re-pigmentation measures using data from the HI-Light trial
Source: Health Qual Life Outcomes. 2023 Aug 10;21:85. doi: 10.1186/s12955-023-02172-4 (PMC10413598; doi:10.1186/s12955-023-02172-4)
Supplement: Supplementary file 6 — Additional file 6: Supplementary Table 3b. Model Parameter Estimates – VNS/RPS Mapping Algorithms. M4: Linear Model; M5: Non-Linear Model; M6: Polynomial Model (VNS M6: Polynomial regression of orders 4, RPS M6: Polynomial regression of orders 3); SE: Standard Error; *statistically significant at 2 sided 5% level or posterior probability of rejecting Null hypothesis (slope=0) is >97.5%. [file 12955_2023_2172_MOESM6_ESM.docx]

**Supplementary Table 5: Model Parameter Estimates – RPS Alava Mapping Algorithm (inclusion of Vitiligo Area/Age)**

|  | **Model** | **Alava** | |
| --- | --- | --- | --- |
|  |  | Estimate (SE) | |
| **RPS Model** | **M7 (Polynomial)** | |  |
|  | Intercept Term | | 0.711 |
|  | Vitiligo Area (Face/Not Face) | | -0.00324 (0.01143) |
|  | RPS | | 0.0119 (0.00871) |
|  | RPS^2^ | | -0.0002145 (0.0001) |
|  | RPS^3^ | | 0.00000118 (<0.001) |
| **RPS Model** | **M8 (Polynomial)** | |  |
|  | Intercept Term | | 0.778 |
|  | Age | | -0.00108 (0.00031) |
|  | RPS | | 0.010608 (0.00865) |
|  | RPS^2^ | | -0.0001915 (0.0001) |
|  | RPS^3^ | | 0.000001059 (<0.001) |

RPS M7/M8: Polynomial regression of orders 3; SE: Standard Error; *statistically significant at 2 sided 5% level or posterior probability of rejecting Null hypothesis (slope=0) is >97.5%
