# Supplementary material for: Estimating health related quality of life effects in vitiligo. Mapping EQ-5D-5 L utilities from vitiligo specific scales: VNS, VitiQoL and re-pigmentation measures using data from the HI-Light trial
Source: Health Qual Life Outcomes. 2023 Aug 10;21:85. doi: 10.1186/s12955-023-02172-4 (PMC10413598; doi:10.1186/s12955-023-02172-4)
Supplement: Supplementary file 7 — Additional file 7: Supplementary Table 4. List of Mapping Algorithms: Van Hout and Alava Crosswalks. M1: Linear Model; M2: Linear Multivariate Model; M3: Bayesian Linear Model ; M4: Linear Model; M5: Non-Linear Model; M6: Polynomial Model (VNS M6: Polynomial regression of orders 4, RPS M6: Polynomial regression of orders 3). TVS : Total VitiQoL Score; items 1 to 16 are the scores from the VitiQoL questions 1 to 16 ; #MV Linear: Multivariate linear for VitiQoL for all 16 items; VNS: Vitiligo Noticeability Scale; RPS: Re-pigmentation; Re-pigmentation Categories: 0-24% [25], 25-49% [50], 50-74% [75], 75-100% [100]. [file 12955_2023_2172_MOESM7_ESM.docx]

**Supplementary Table 6: Cross Validation Results (VNS, RPS) – Final Models : Model M2**

|  | **Van Hout Crosswalk Utilities** | | **Alava Crosswalk Utilities** | |
| --- | --- | --- | --- | --- |
|  | **VNS M6** | **RPS M6** | **VNS M6** | **RPS M6** |
| **Predicted Mean (SE)** | 0.908 (0.0005) | 0.907 (0.0005) | 0.894 (0.0004) | 0.899 (0.0006) |
| **Observed Mean (SE)** | 0.900 (0.007) | 0.905 (0.007) | 0.894 (0.007) | 0.901 (0.007) |
| **Mean Difference (Observed vs Predicted)** | -0.008 | -0.002 | 0.0008 | 0.002 |
|  |  |  |  |  |
| **Predicted QALY (SE)** | 1.593 (0.005) | 1.587 (0.007) | 1.565 (0.004) | 1.575 (0.008) |
| **Observed QALY (SE)** | 1.576 (0.039) | 1.506 (0.082) | 1.561 (0.042) | 1.535 (0.055) |
| **QALY Difference (Observed vs Predicted)** | -0.017 | -0.081 | -0.004 | -0.040 |

M6: Polynomial Model (VNS M6: Polynomial regression of orders 4, RPS M6: Polynomial regression of orders 3). Observed and predicted means generated from randomly selected datasets for cross validation purposes, two separate datasets were used for the VNS M6 predictions and RPS M6 predictions. Difference: Observed – Predicted. QALY: Quality Adjusted Life Year; SE: Standard Error; VNS: Vitiligo Noticeability Scale; RPS: Re-pigmentation Score; QALY estimates derived from baseline, month 9 and month 21 data.
