# Supplementary figures and images for: Estimating health related quality of life effects in vitiligo. Mapping EQ-5D-5 L utilities from vitiligo specific scales: VNS, VitiQoL and re-pigmentation measures using data from the HI-Light trial
Source: Health Qual Life Outcomes. 2023 Aug 10;21:85. doi: 10.1186/s12955-023-02172-4 (PMC10413598; doi:10.1186/s12955-023-02172-4)

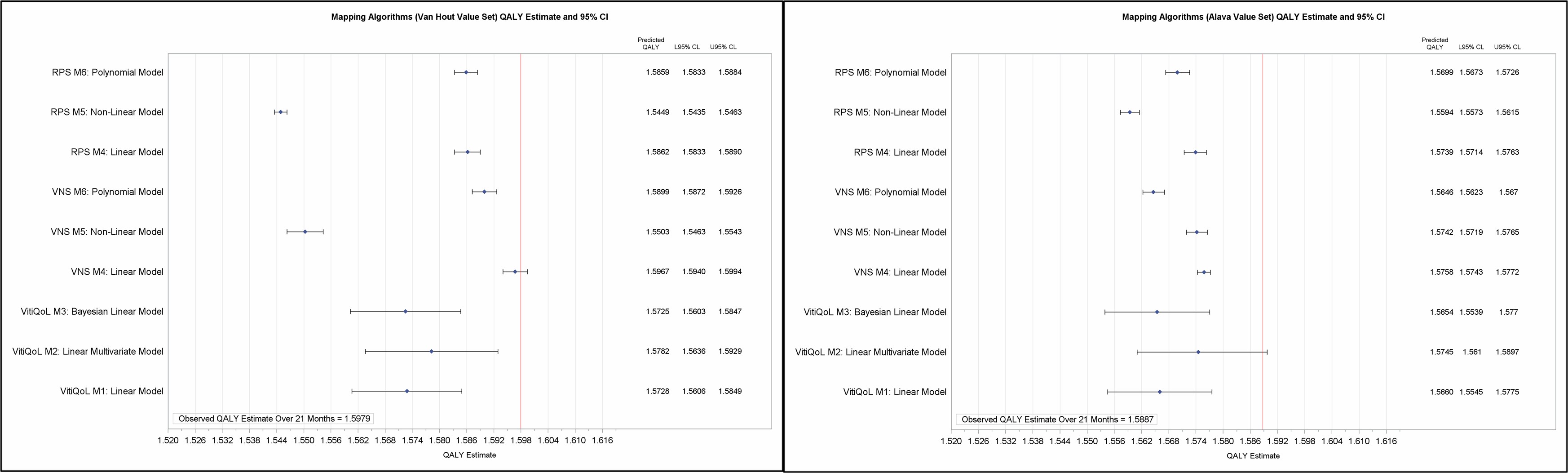

Supplement: Supplementary file 8 — Additional file 8: Supplementary Table 5. Model Parameter Estimates – RPS Alava Mapping Algorithm (inclusion of Vitiligo Area/Age). RPS M7/M8: Polynomial regression of orders 3; SE: Standard Error; *statistically significant at 2 sided 5% level or posterior probability of rejecting Null hypothesis (slope=0) is >97.5%. [file 12955_2023_2172_MOESM8_ESM.jpg]

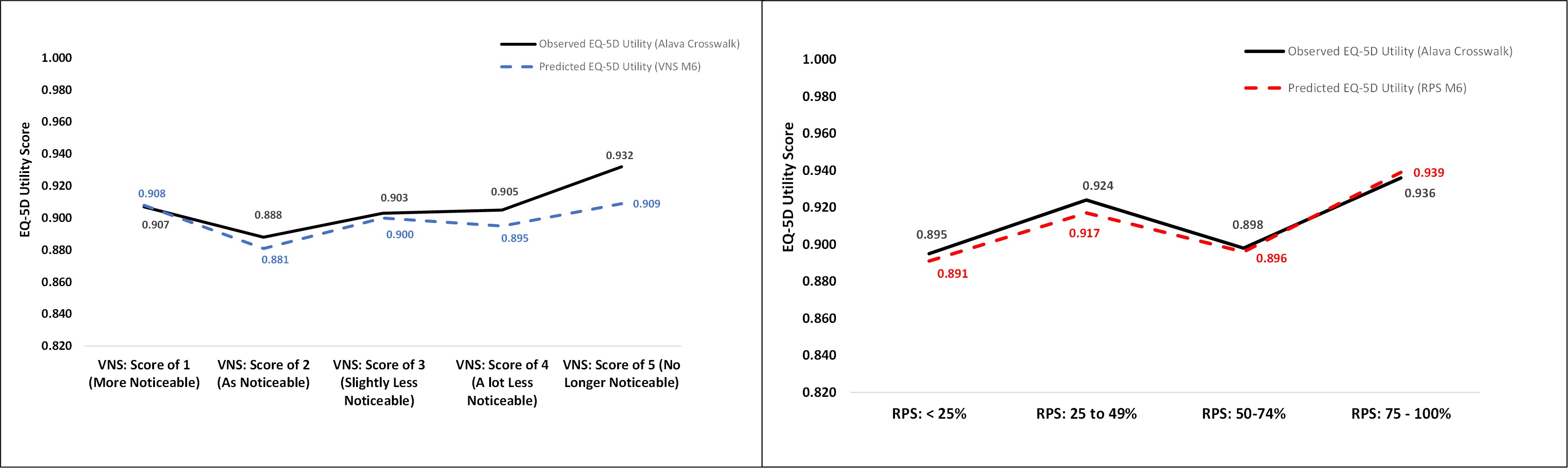

Supplement: Supplementary file 9 — Additional file 9: Supplementary Table 6. Cross Validation Results (VNS, RPS) – Final Models. M6: Polynomial Model (VNS M6: Polynomial regression of orders 4, RPS M6: Polynomial regression of orders 3). Observed and predicted means generated from randomly selected datasets for cross validation purposes, two separate datasets were used for the VNS M6 predictions and RPS M6 predictions. Difference: Observed – Predicted. QALY: Quality Adjusted Life Year; SE: Standard Error; VNS: Vitiligo Noticeability Scale; RPS: Re-pigmentation Score; QALY estimates derived from baseline, month 9 and month 21 data. [file 12955_2023_2172_MOESM9_ESM.jpg]
